# Research funders’ roles and perceived responsibilities in relation to the implementation of clinical research results: a multiple case study of Swedish research funders

**DOI:** 10.1186/s13012-015-0290-5

**Published:** 2015-07-17

**Authors:** Anders Brantnell, Enrico Baraldi, Theo van Achterberg, Ulrika Winblad

**Affiliations:** Department of Public Health and Caring Sciences, Uppsala University, Uppsala, Sweden; Department of Industrial Engineering and Management, Uppsala University, Uppsala, Sweden; Centre for Health Services and Nursing Research, KU Leuven, Leuven, Belgium

**Keywords:** Quality improvement, Implementation responsibility, Support of research, Research funder roles

## Abstract

**Background:**

Implementation of clinical research results is challenging, yet the *responsibility* for implementation is seldom addressed. The process from research to the use of clinical research results in health care can be facilitated by *research funders*. In this paper, we report the roles of ten Swedish research funders in relation to implementation and their views on responsibilities in implementation.

**Findings:**

Ten cases were studied and compared using semi-structured interviews. In addition, websites and key documents were reviewed. Eight *facilitative* roles for research funders in relation to the implementation of clinical research results were identified. Three of them were common for several funders: “Advocacy work,” “Monitoring implementation outcomes,” and “Dissemination of knowledge.” Moreover, the research funders identified six different actors responsible for implementation, five of which belonged to the healthcare setting. Collective and organizational responsibilities were the most common forms of responsibilities among the identified actors responsible for implementation.

**Conclusions:**

The roles commonly identified by the Swedish funders, “Advocacy work,” “Monitoring implementation outcomes,” and “Dissemination of knowledge,” seem feasible facilitative roles in relation to the implementation of clinical research results. However, many actors identified as responsible for implementation together with the fact that collective and organizational responsibilities were the most common forms of responsibilities entail a risk of implementation becoming no one’s responsibility.

**Electronic supplementary material:**

The online version of this article (doi:10.1186/s13012-015-0290-5) contains supplementary material, which is available to authorized users.

## Background

Each year, research funders allocate large resources to universities which study and develop medical treatments. Implementation is in turn the process whereby the output of these studies is introduced in health care to improve patient care. The output implemented consists of new treatments, often, in the form of clinical guidelines, which in turn are based on state-of-the-art reviews [1]. Such reviews are often a combination of several clinical research studies summarized into recommendations, but in the essence, the output consists of clinical research results, i.e., results from research that involves human subjects or specimen in developing and testing new treatments to improve patient care [2]. However, the implementation of clinical research results is inadequate, and there is a wide knowledge-practice gap implying that patients do not receive optimal care [3]. Much focus to date has been on healthcare practitioners’—the knowledge users—role in implementation [4]. Factors such as healthcare practitioners’ existing attitudes and skills have been identified to hinder the behavior change needed to adopt the new treatments [5]. In a comprehensive review, Flottorp et al. identified 57 barriers and facilitators for implementation at seven different levels, many of which had to do not only with healthcare practitioners but also with the evidence/clinical research results, the providing organization, and the surrounding society [6]. Consequently, implementation is a complex process that aims to change the behavior of healthcare practitioners in order to improve patient care. However, as implementation takes place in a wider socio-economic-political context, the science of implementation can be developed further if alternative approaches are utilized to study the knowledge-practice gap [7].

Such an alternative line of enquiry consists of expanding the focus from healthcare providers to also studying the effects of including other actors who, although more distant from practice, can influence the implementation of clinical research results. More precisely, we argue that *research funders* (hereafter funders), who decide over resource allocation, may play an important role in the implementation of clinical research results by facilitating the steps leading to implementation and implementation itself. Traditionally, funders’ roles consist of (a) receiving grant applications, (b) evaluating them, (c) funding the most suitable ones, and (d) evaluating research outputs [8]. Thus, funders have traditionally not been interested in the practical effects of clinical research results [9]. However, new funder roles are emerging, pushed forward by the funders of funders—often governments—concerned by the fact that resources invested in research are not matched by improvements in public health [10, 11]. These roles can be termed *facilitative* and are defined in this paper as activities that go beyond the four traditional tasks above (roles a–d) and deal with activities that are relevant for implementation. Previous studies have recognized several facilitative roles for funders: involvement in implementation of innovations in health care [12], advocating for the use of research results [11], managing implementation programs [10], creating interaction between researchers and research users [13, 14], demanding that researchers submit an implementation plan together with their grant application [12], and disseminating research findings [15].

Earlier studies often limit themselves to cases of single funders [10, 11, 13, 15], although a couple of exceptions exist, which are international mapping studies focusing on health research funders efforts and interest to contribute to improved the use of research results in practice [16, 17]. These studies identified differences on how various funders defined their efforts and the extent to which they engaged in these activities [16, 17]. Despite this, there is a dearth of studies that more systematically investigate the perceptions of funders and their facilitative roles in relation to implementation.

Although the focus in this paper is on funders, it should be highlighted that the users of clinical research results are the healthcare practitioners and in some cases, implementation of new clinical research results might not be appropriate, e.g., if the evidence base is too thin [3] or in the case the old treatments need to be de-implemented first [18]. However, even if the clinical research results are convincing and clearly point at a need for implementation, implementation does not happen automatically. Implementation needs initiators, facilitators, and persons responsible for it. To this end, the emerging facilitative roles of funders require that the funders have knowledge in implementation [19] and an understanding of who is responsible for implementation [10]. Responsibility for implementation of clinical research results is seldom made explicit and problematized, and funders’ knowledge about implementation responsibilities is, to the best of our knowledge, not addressed. Against this background, our aims are (1) to identify the facilitative roles of ten Swedish funders of clinical research in relation to implementation and (2) to analyze the funders’ views about implementation responsibilities.

## Findings

### Theoretical starting point to study responsibilities

The responsibility for implementation is a problematic issue, as suggested by the “problem of many hands.” This theory is presented below and highlights a set of difficulties related to implementation. In complex healthcare organizations, where many different hierarchical levels and actors participate, the “problem of many hands” implies that it might be difficult to state who actually is responsible for implementation [20]. More specifically, the responsibility might be divided between or found at two different levels: *the organizational* [21] and *the personal levels* [22]. Organizational responsibility means that the organization as a whole takes responsibility whereas personal responsibility can be divided into three strains: (a) “Hierarchical,” whereby it is the highest-ranked person—the director of the healthcare organization—who should take responsibility, (b) “Collective,” whereby all involved persons—e.g., a team of healthcare professionals—should take responsibility, and (c) “Individual,” whereby each person—an individual healthcare professional—takes responsibility. Consequently, the problem of “many hands” indicates that it might be difficult to state who or which collective actually is responsible and that the different levels of responsibilities might hinder responsibility taking, for instance, collective responsibility might entail that implementation becomes no one’s responsibility [23]. In this paper, the “problem of many hands” guided the research concerning implementation responsibilities and was used to analyze findings regarding responsibilities.

## Methods

Ten cases were studied and compared with the help of 18 semi-structured interviews. The ten cases were selected in order to capture a representative sample of Swedish funders funding clinical research in terms of the size of funding, geographical scope, and type of funders (Table 1).

**Table 1:** Characteristics of the selected research funders

Funders	Function and mission	Type of research funded	Resources^a^	Geographical scope and type
1. The Education Council	Prepares decisions for funding by government	All main types of research (medical, social science, technology, natural sciences)	370 million Euros	National and public
	Debates issues regarding allocation of public research funds			
2. The Research Council for Medicine and Health	Funds basic and applied research	Medical (including clinical) odontological, pharmacological, care science^b^	32 million Euros	National and public
	Specific focus on medicine and health			
3. Sweden’s Innovation Agency (Vinnova)	Funds applied research	Clinical, biomedical, health services, pharmacological	30 million Euros	National and public
	Health is one of the focus areas			
4. The Vårdal Foundation	Funds applied research	Care science	4 million Euros	National and private
	Specific focus on health			
5. The Swedish Childhood Cancer Foundation	Funds basic and applied research	Clinical, epidemiological, biological, care science, psychosocial	14 million Euros	National and private
	Specific focus on abolishment of childhood cancer			
6. The Swedish Cancer Society	Funds basic and applied research	Clinical, epidemiological, pre-clinical, translational, care science	40 million Euros	National and private
	Specific focus on abolishment of cancer			
7. The County Council of Uppsala	Funds applied research	Clinical	18 million Euros	Local and public
	Specific focus on clinical research			
8. The County Council of Västerbotten	Funds applied research	Clinical	15 million Euros	Local and public
	Specific focus on clinical research			
9. The County Council of Stockholm	Funds applied research	Clinical	41 million Euros	Local and public
	Specific focus on clinical research			
10. Region Skåne^c^	Funds applied research	Clinical	29 million Euros	Local and public
	Specific focus on clinical research			

^a^The figures for the Education Council indicate how much resources they indirectly decide over, and the figures for other funders indicate how much they directly allocate to clinical research on an annual basis. Funds from funders 2–3 and 7–10 are included in the resources that the Education Council decides over. The figures are based on elaboration of statistics between 2008 and 2012

^b^Care science is mostly a Swedish definition and includes different disciplines such as nursing science, occupational therapy, psychosocial research, physiotherapy, and rehabilitation

^c^Region Skåne is officially a region but has in general the same functions and tasks as a County Council

The ten funders’ represent three different levels in the research funding system: (1) national public funding, (2) national, private non-profit funding, and (3) local public funding (see Additional file 1).

The same interview guide was used with all respondents with minor adaptations to suit funders’ organization and respondent’s position (see Additional file 2). No specific definition of the primary concepts discussed in this paper concerning roles and responsibilities was provided to respondents as exploring their opinions, instead of forcing them to use specific concepts, was preferred. In order to diminish bias, methodological triangulation [24] was used through data collected from websites, annual reports, and goal statements (Table 2).

**Table 2:** Documents and sources reviewed in order to enhance the rigor of the study

Funder	Type	Description
The Research Council for Medicine and Health	Website	www.vr.se/amnesomraden/amnesomraden/medicinochhalsa.4.12fff4451215cbd83e4800020161.html
The Research Council for Medicine and Health	Goal statement	Program statement 2013-2016
Sweden’s Innovation Agency (Vinnova)	Website	www.vinnova.se/en/
Sweden’s Innovation Agency (Vinnova)	Annual report	Years 2012 and 2013
The Vårdal Foundation	Website	www.vardal.se/topp-meny/in-english/
The Vårdal Foundation	Annual report	Years 2012 and 2013
The Swedish Childhood Cancer Foundation	Website	www.barncancerfonden.se/In-english/
The Swedish Childhood Cancer Foundation	Annual report	Years 2012 and 2013
The Swedish Cancer Society	Website	www.cancerfonden.se/sv/Information-in-English/
The Swedish Cancer Society	Annual report	Years 2012 and 2013
All County Councils	Goal statement	Contract^a^ between the Swedish government and certain County Councils concerning cooperation about education of physicians, medical research, and development of health care
The County Council of Uppsala	Goal statement	Regional contract between the Uppsala University and the County Council of Uppsala concerning cooperation about education of physicians, medical research, and development of health care
The County Council of Västerbotten	Goal statement	Regional contract between the Umeå University and the County Council of Västerbotten concerning cooperation about education of physicians, medical research, and development of health care
The County Council of Stockholm	Goal statement	Regional contract between the County Council of Stockholm and the Karolinska Institutet concerning cooperation about education of physicians, medical research, and development of health care
Region Skåne	Goal statement	Regional contract between the Region Skåne and the Lund University concerning cooperation about education of physicians, medical research, and development of health care

^a^This contract and its regional versions between County Councils and Universities are often called ALF contracts where ALF stands for “contract between the Swedish government and certain County Councils concerning cooperation about education of physicians, medical research, and development of health care”

The data was analyzed using an abductive approach [25], which is a suitable approach when one aims to study phenomena in their context starting from existing theory [26]. Both “within-cases” and “across-cases” analyses [27] were conducted. For a more detailed description of the methods used in this study, see Additional file 3.

### Roles and responsibilities in implementation

The three tables (3–5) below indicate our findings showing which level the funder represents (national public, national private, or local public). The three tables allow for following the analysis both within the same level of funders if read vertically and between different levels of funders if read horizontally.

### Funders roles in implementation

Regarding funders’ facilitative roles in relation to implementation, two common roles for two different funding levels were identified: “Advocacy work” and “Monitoring implementation outcomes” (Table 3). “Advocacy work” was mentioned by one national public and three national private funders. All four defined the role “Advocacy work” in a similar manner but worked with advocacy from different standpoints, for instance, the national public funder stated that their role was to communicate among decision-makers that implementation is important, whereas the national private funder attempted to convince decision-makers to invest in implementation and also saw their role as educating the general public.

**Table 3:** Research funder (*N* = 10) roles in relation to the implementation of clinical research results

Research funder^a^ roles	Definition of funder roles	National public (*N* = 3)	National private (*N* = 3)	Local public (*N* = 4)	Total funding levels^b^
1. Advocacy work	Aims to create awareness and increase knowledge among decision-makers at different levels through different means about feasibility and costs of implementation of clinical research results	1 funder^c^	3 funders		4 funders
2. Monitoring implementation outcomes	Follows-up, evaluates, and reports the results of implementation of clinical research results regarding outcomes and costs		2 funders	1 funder	3 funders
3. Dissemination of knowledge	Through different means spread information about clinical research results		3 funders		3 funders
4. Work actively towards implementation	Is engaged and takes responsibility during the whole research process from research start to receiving of output and is prepared to adjust the plans during the process		1 funder		1 funder
5. Create conditions for implementation through legislation in implementation related issues	Enables for the healthcare professionals to get access to the state of the art knowledge by establishing organizations that can produce guidelines	1 funder			1 funder
6. Stimulate collaboration between researchers and industry	Organizes research projects which demand involvement of private companies that are going to use the output of the project	1 funder			1 funder
7. Educate healthcare personnel and parents to patients	Organizes education of healthcare personnel and parents to children suffering from cancer		1 funder		1 funder
8. Create structures for organized introduction	Redesigns health care providing organizations so that they are capable of integrating new clinical research results in healthcare practice			1 funder	1 funder
Total funder roles^d^		3 roles	5 roles	2 roles	

^a^Multiple roles per funder allowed

^b^Summarizes the amount of funders supporting each role horizontally across funding levels. NB: the sum of these totals is higher than 10, due to multiple answers allowed per funder

^c^Indicates the amount of funders supporting each role

^d^Summarizes the amount of roles vertically for funders within each funding level

Two national private funders and one local public funder considered that “Monitoring implementation outcomes” is an important part of their work in relation to implementation. The national private funders had a structured way to monitor implementation outcomes either through a yearly published report describing the research results and their implementation or through an updated register about treatments that had been implemented and the outcomes for patients. “Dissemination of knowledge” was a role mentioned only by the three private funders who described this in similar terms, i.e., as something that goes beyond sending out newsletters and publishing results on the internet. In addition to these three common roles, the ten funders mentioned five other roles: “Work actively towards implementation,” “Create conditions for implementation through legislation in implementation related issues,” “Stimulate collaboration between researchers and industry,” “Educate healthcare personnel and parents,” and “Create structures for organized introduction.” All of these eight roles, except partly the role “Monitoring implementation outcomes,” were presented as roles the funders currently already fulfilled as the funders illustrated these with their concrete activities. The local public funder who mentioned the role “Monitoring implementation outcomes” did not illustrate this role with specific activities and thus seemed more of a potential role the funder could or should fulfill. On the other hand, the two national private funders who mentioned the role “Monitoring implementation outcomes” explained the role through concrete activities.

### Responsibility for implementation?

The funders identified six different actors responsible for implementation. Table 4 provides the list of these actors.

**Table 4:** Who is responsible for implementation of clinical research results and do these actors take responsibility?

Actors identified and perceived responsibility taking^a^	National public (*N* = 3)	National private (*N* = 3)	Local public (*N* = 4)	Total funding levels^b^
1. County Councils	1 funder^c^ Somewhat^d^ (1 funder)	1 funder No (1 funder)	1 funder Somewhat (1 funder)	3 funders Somewhat (2 funders), No (1 funder)
2. Head of hospital units		1 funder Yes (1 funder)	2 funders Yes (1 funder), Somewhat (1 funder)	3 funders Yes (2 funders), Somewhat (1 funder)
3. Healthcare system	1 funder Somewhat (1 funder)			1 funder Somewhat (1 funder)
4. Medical practitioners together with County Councils	1 funder Somewhat (1 funder)			1 funder Somewhat (1 funder)
5. Research funders together with the researcher		1 funder Yes (1 funder)		1 funder Yes (1 funder)
6. Hospital leadership			1 funder Yes (1 funder)	1 funder Yes (1 funder)
Total funders^e^	3 actors Somewhat (3 funders)	3 actors Yes (2 funders), No (1 funder)	3 actors Somewhat (2 funders), Yes (2 funders)	10 funders Somewhat (5 funders), No (1 funder), Yes (4 funders)

^a^One answer per funder allowed

^b^Summarizes the total amount of funders suggesting each responsible actor across funding levels (*N* = 10) and the responsibility alternatives across funding levels where the possible alternatives are “Yes,” “To a certain degree,” and “No.” The amount of funders, regarding each responsibility alternative, is given in brackets

^c^The amount of funders within funding levels suggesting each responsible actor is indicated, followed by views on responsibility taking and the amount of funders indicating each view, which is given in brackets

^d^Somewhat stands for “To a certain degree”

^e^Summarizes the amount of identified actors by funders and responsibility alternatives vertically for funders within each funding level. The amount of funders, regarding each responsibility alternative, is given in brackets

The “County Councils” (three funders from three different levels), followed by the “Head of hospital units” (three funders from two different levels), were the actors most often pinpointed as responsible for implementation. The key role of the County Councils was further underlined by the fact that one more funder viewed them as responsible *together with* other actors, namely medical practitioners. The national public funders agreed in general that it is the County Councils, alone or in concert with other actors, who are responsible for implementation. Similarly, national private funders leaned towards the County Councils and the “Head of hospital units” as responsible actors, with the exception of one private funder who stated that it is the research funders together with researchers who are responsible for implementation. Also, the local public funders considered that the County Councils or actors within the County Councils are responsible for implementation. Consequently, the majority of the funders considered that the responsibility for implementation is located in the healthcare setting and preferably shouldered by the County Councils or actors within County Councils.

### Do the identified actors take responsibility for implementation?

The majority of the funders who considered that County Councils are responsible for implementation stated that these take responsibility for implementation of clinical research results “To a certain degree.” For this incomplete responsibility taking, two explanations were proposed: (1) the County Councils’ systems for bringing clinical research results into practice are not really clear and (2) the County Councils’ focus on saving lives and cost control do not always ease implementation of new findings. A more negative view was taken by one national private funder who considered that the County Councils are not organizationally capable of taking such responsibility. The majority of the funders selecting the option “Head of hospital units” considered that this actor takes responsibility, whereas a minority stated that this actor takes responsibility “To a certain degree.” In general, five funders considered that the actors they identified take responsibility “To a certain degree,” four funders thought that the identified actors take responsibility and one funder considered that the identified actor does not take responsibility (Table 4). As shown in Table 5, half of the funders considered that *no one else should take responsibility* for implementation, followed by three funders who thought that someone else should take responsibility “To a certain degree” and two funders who considered that *someone else* should take responsibility. A more detailed description of the findings can be found in Additional file 4.

**Table 5:** Should someone else take responsibility for implementation of clinical research results?

Should someone else take responsibility?^a^	National public (*N* = 3)	National private (*N* = 3)	Local public (*N* = 4)	Total^b^
Yes	1 funder^c^		1 funder	2 funders
To a certain degree	1 funder		2 funders	3 funders
No	1 funder	3 funders	1 funder	5 funders

^a^Only one answer per funder allowed

^b^Indicates the total amount of funders across funding levels supporting each option where the possible alternatives are “Yes,” “To a certain degree,” and “No” (*N* = 10)

^c^Indicates the amount of funders within funding levels supporting each option

## Discussion

“Advocacy work,” “Monitoring implementation outcomes,” and “Dissemination of knowledge” are the three most popular roles. “Advocacy work,” supported by two funding levels is an acknowledged role for funders [11]. “Monitoring implementation outcomes,” supported also by two funding levels, is about activities occurring *after* implementation and is not reported in previous research regarding funders but is well documented in policy and program implementation contexts [28–30]. Also, “Dissemination of knowledge,” supported by only three private national funders, is reported in previous research [15, 17]. The majority of the funders consider that the actual responsibility for implementation lies in the healthcare setting. However, five funders express that the actors they identify take responsibility “To a certain degree,” which implies that although the funders identify the actors responsible for implementation they are not entirely convinced that the identified actors *actually* take responsibility. Despite this, five out of ten funders consider that no one else should take responsibility for implementation of clinical research results and only two out of ten express that someone else should do that. The fact that funders from the three funding levels together propose five different County Council-related actors as responsible for implementation is not surprising as the “problem of many hands” suggests that in complex organizations, it might be difficult to state who actually is responsible [20]. Furthermore, the majority of the funders refer either to a group of actors (actor 6 in Table 4), to organizations (actors 1 and 3 in Table 4), or to a combination of actors and organizations (actor 4 in Table 4) which entails either collective or organizational responsibility [23]. Prevalence of collective and organizational responsibility together with the fact that it is difficult for funders to identify actors responsible for implementation indicates that in complex healthcare organizations, responsibility for implementation risks becoming no one’s responsibility (see Additional file 5 for a detailed discussion).

### Study limitations

This study utilized an explorative approach in studying funders’ roles and perceived responsibilities in relation to implementation, as these issues were assumed to be rather new for many funders. Consequently, it was not deemed appropriate to send out a questionnaire to all clinical research funders that could have opened up for a quantitative analysis. An alternative method could have been an in-depth study of one or few implementation cases, focusing on the roles of funders and other actors in action. This method could have captured the mechanisms and roles of all involved actors (funders, actors responsible for implementation, and implementers) and their perceptions about their own and the funders’ roles but would not have provided a representative picture of the Swedish research funding system. Instead, the decision to obtain a complete picture of clinical research funders in Sweden, using a representative sample as for resources and geographical scope, was chosen. Moreover, the selected approach covered three important funding levels.

### Conclusions and implications

“Advocacy work,” “Monitoring implementation outcomes,” and “Dissemination of knowledge” are common roles for Swedish research funders. “Monitoring implementation outcomes” is not reported in previous research. The Swedish research funders identify six different actors responsible for implementation, indicating difficulties to state the actors responsible for implementation in accordance with the “problem of many hands.” Moreover, the existence of a “problem of many hands” is supported by the finding that the prevalent form of responsibility is either collective or organizational responsibility. These findings together imply that in complex healthcare organizations, implementation risks become no one’s responsibility. This finding has bearing not only for healthcare providers when they are planning and conducting implementation but also for funders who wish to facilitate implementation in different ways, as well as for funders of funders who allocate resources to clinical research aiming to improve public health. Our findings have implications also for implementation researchers demonstrating the relevance of studying funders’ roles and responsibility issues in relation to implementation and thus justifying the necessity of a broader scope in order to understand implementation and the steps leading to it.

## Additional files

Additional file 1:
**Description of the three funding levels.** This file describes the selected funders and connects them with the funding levels. Additional file 2:
**Summarized interview guide.** This file presents a summarization of the questions posed to the respondents. Additional file 3:
**Detailed description of the methods.** This file describes more thoroughly the methods applied. Additional file 4:
**Detailed description of the findings.** This file describes more thoroughly the findings. Additional file 5:
**Detailed discussion of the findings.** This file discusses more thoroughly the findings.
