# Supplementary material for: Research funders’ roles and perceived responsibilities in relation to the implementation of clinical research results: a multiple case study of Swedish research funders
Source: Implement Sci. 2015 Jul 17;10:100. doi: 10.1186/s13012-015-0290-5 (PMC4506440; doi:10.1186/s13012-015-0290-5)
Supplement: Additional file 1: — Description of the three funding levels. This file describes the selected funders and connects them with the funding levels. [file 13012_2015_290_MOESM1_ESM.docx]

| **Additional file 1. Description of the three funding levels** | |
| --- | --- |
| **Level** | **Description** |
| **National public level** | The Education Council (*Utbildningsutskott*) is the first national public level funder and functions as a preparatory organ for the Swedish parliament in questions of education and research. The members of the Education Council reflect the party balance in the Swedish parliament: thus, the Education Council represents a miniature version of the actual parliament, so that the opinions of the Education Council and the parliament are not likely to differ. The Education Council is not a direct funder, but handles budget proposals from the government and is, therefore, an influential actor in the allocation of clinical research funds to various central and local funding agencies. The funds consist mainly of “ALF” resources (allocated to the County Councils), resources allocated to the university faculties, funds allocated to the Research Council for Medicine and Health, and resources to the Swedish Innovation Agency.  The Research Council for Medicine and Health (*Rådet för medicin och hälsa*), is the second national public level funder and finances both basic and applied research.  Sweden’s Innovation Agency (Vinnova), is the third national public level funder and funds only applied research and demands always co-funding from contributing organizations. |
| **National, private non-profit level** | The Vårdal Foundation (*Vårdalstiftelsen*) is the first national, private non-profit level funder, and it is a foundation established with the help of public resources in the 1990s, but operating now independently in funding applied research.  The Swedish Childhood Cancer Foundation (*Barncancerfonden*) and the Swedish Cancer Society (Cancerfonden) are the second and third national, private non-profit level funders. Both these are funded by private donors and they support both basic and applied research. They are the biggest funders in their respective fields in Sweden. |
| **Local public level** | County Councils represent the local public level funders. All County Councils use two main types of resources to fund clinical research and healthcare development: “R&D resources”, covered by local taxes, and “ALF” resources coming from the central government, based on a decision at the Swedish parliament and its Education Council. The focus, in this paper, is on “ALF” resources as these are mainly used to fund clinical research, whereas the R&D resources are used to fund healthcare development in general. Every County Council has created a specific system for coordination and allocation of “ALF” resources.  The first local public level funder is the County Council of Uppsala where it is the “ALF Board” (*ALF styrelsen*), composed of representatives from Uppsala University and politicians from the County Council who make the allocation decisions.  The second local public level funder is the County Council of Västerbotten where the Coordination Board (*Samverkansstyrelsen*), composed of representatives from the University of Umeå and politicians from the County Council, decide over the allocation of resources.  The third local public level funder is the County Council of Stockholm, where it is the “County Council Boards Working Committee” (*Landstingsstyrelsens arbetsutskott*) consisting of politicians who decide over the allocation of resources. The fourth local public level funder is the Region of Skåne (formally not a County Council, but operating in a similar manner) who allocates the resources. These four local public funders are part of a bigger organization, County Council, who is responsible for healthcare delivery. |
