# Supplementary material for: Research funders’ roles and perceived responsibilities in relation to the implementation of clinical research results: a multiple case study of Swedish research funders
Source: Implement Sci. 2015 Jul 17;10:100. doi: 10.1186/s13012-015-0290-5 (PMC4506440; doi:10.1186/s13012-015-0290-5)
Supplement: Additional file 2: — Summarized interview guide. This file presents a summarization of the questions posed to the respondents. [file 13012_2015_290_MOESM2_ESM.docx]

| **Additional file 2. Summarized interview guide** |
| --- |
| 1. What is your name? |
| 2. What is your profession? |
| 3. What is your job content? |
| 4. What is your position in the organization? |
| 5. How long have you had this position? |
| 6. How long have you been involved in the work of the organization? |
| 7. What is the assignment and goals of the funding organization regarding clinical research funds? |
| 8. What does the organization do to achieve the goals with the clinical research funds? |
| 9. Which are the responsibilities of the organization regarding the clinical research funds? |
| 10. What do you think about these responsibilities? |
| 11. Which role does the organization have in implementation of clinical research results? |
| 12. Is there someone who is responsible for implementation of clinical research results? Who in that case? |
| 13. Do you think that this actor takes responsibility for implementation of clinical research results? |
| 14. Should someone else take responsibility? |
| 15. Is there someone else who I should interview and who would be the key person in this context and also member of the funding body? |
