# Supplementary material for: Research funders’ roles and perceived responsibilities in relation to the implementation of clinical research results: a multiple case study of Swedish research funders
Source: Implement Sci. 2015 Jul 17;10:100. doi: 10.1186/s13012-015-0290-5 (PMC4506440; doi:10.1186/s13012-015-0290-5)
Supplement: Additional file 3: — Detailed description of the methods. This file describes more thoroughly the methods applied. [file 13012_2015_290_MOESM3_ESM.docx]

# Additional file 3. Detailed description of the methods

## Study design

A multiple explorative case study [1] among Swedish research funders funding clinical research was conducted. 10 cases were selected in order to capture a representative sample of Swedish research funders funding clinical research in terms of size of funding, geographical scope and type of funders.

## Sample

The 10 selected funders (Table 1) cover approximately 60% of all clinical research funding in Sweden, which yearly amounts to about Euro730 million, with the central public level allocating Euro370 million, the private organizations allocating Euro110 million, the local public level granting Euro130 million, and other funders (including private companies and the EU) providing Euro120 million [2]. The funders selected for this study represent three different levels in the research funding system: 1) national public funding, 2) national, private non-profit funding, and 3) local public funding. They vary in geographical scope as four out of seven local public funders, who fund clinical research in Sweden, were included. The three levels of funders accordingly act at different distances from healthcare, with national public funders being farthest away and local public funders being closest to healthcare.

At the 10 selected funders, respondents from the highest decision-making bodies were chosen. Admittedly several levels of decision making exist at each funder, but the selected respondents make the final decisions on funds allocation. The respondents were included based on seniority, experience and knowledge of clinical research.

The selected funders represent three important levels of the Swedish research funding system. Appendix 1 gives a short description of the three funding levels.

## Data collection

Two semi-structured, face-to-face, interviews per funder were conducted, except in two cases (Vinnova and Region Skåne) where there was only one respondent who fulfilled the inclusion criteria. The 18 respondents were identified by the research team and every approached respondent participated. In addition to this, the relevance of the respondents was validated by means of asking the respondents to identify key respondents within their organization. One researcher conducted all the interviews. The same interview guide was used with all respondents with minor adaptations to funders’ organization and respondent’s position. No specific definition of the primary concepts discussed in this paper concerning roles and responsibilities was provided to respondents as exploring their opinions, instead of forcing them to use specific concepts, was preferred. The interviews were recorded and transcribed verbatim. The data was collected between April and September 2012. In order to diminish bias, methodological triangulation [3] was used through data collected from websites, annual reports and goal statements, which were reviewed in order to find out how the funders described their official roles and to which degree responsibility issues for implementation were discussed by the funders (Table 2).

## Analysis

The data was analyzed using an abductive approach [4, 5], which is a suitable approach when one aims to study phenomena in their context starting from existing theory [6]. This approach, which combines induction and deduction, allowed the initiation of this study with meaningful pre-determined concepts (about roles and responsibilities). The data collection was influenced by the theoretical considerations regarding research funders’ roles and, for implementation responsibility, the “problem of many hands”. Nonetheless, the answers of the individual respondents were coded inductively before forming the categories. In case the respondents within the same case had differing views these were discussed within the research team and a coding scheme was drafted which facilitated the balancing of these differing views and allowed arriving at the level of cases (n=10). As the core concepts were not defined for the respondents, every category was carefully analyzed and the categories that did not match the definitions (about roles and responsibilities) used in this paper were not included in the results.

Both a “within-cases” and an “across-cases” analysis [7] was conducted comparing the perceptions within the same level of funders and between different levels of funders, searching for similarities and differences. The primary focus was on categories that were common for several funders, but even categories, mentioned by only one funder, were included when this was deemed to add something to the overall findings. The coding schemes were discussed and evaluated by the research team in order to enhance their clarity and validity. The tables were constructed by one researcher and checked for validity by another researcher.

The study protocol was submitted to the Regional Ethical Review Board in Uppsala, who stated that ethical approval was not required for this study.

# References

1. Creswell JW: *Qualitative Inquiry and Research Design: Choosing Among Five Approaches.* 2nd ed. Thousand Oaks: SAGE; 2007.

2. SOU: *Världsklass! Åtgärdsplan för den kliniska forskningen. Delbetänkande av utredningen av den kliniska forskningen. SOU 2008:7*. Stockholm: Utbildningsdepartementet; 2008.

3. Denzin N: *The Research Act in Sociology: A Theoretical Introduction to Sociological Methods.* London: Butterworths; 1970.

4. Thomas G: **Doing Case Study: Abduction Not Induction, Phronesis Not Theory**. *Qual Inq* 2010, **16**:575–582.

5. Dubois A, Gadde L-E: **Systematic combining: an abductive approach to case research**. *J Bus Res* 2002, **55**:553–560.

6. Shah R, Goldstein SM, Unger BT, Henry TD: **Explaining Anomalous High Performance in a Health Care Supply Chain**. *Decis Sci* 2008, **39**:759–789.

7. Yin RK: *Case Study Research: Design and Methods.* 3rd ed. Thousand Oaks: SAGE; 2003.
