# Supplementary material for: Research funders’ roles and perceived responsibilities in relation to the implementation of clinical research results: a multiple case study of Swedish research funders
Source: Implement Sci. 2015 Jul 17;10:100. doi: 10.1186/s13012-015-0290-5 (PMC4506440; doi:10.1186/s13012-015-0290-5)
Supplement: Additional file 4: — Detailed description of the findings. This file describes more thoroughly the findings. [file 13012_2015_290_MOESM4_ESM.docx]

# Additional file 4. Detailed description of the findings

The findings indicate which level the funder represents (national public, national private or local public), and the three tables featuring the findings allow for following the analysis both within the same level of funders if read vertically, and between different levels of funders, if read horizontally.

## Research funder roles in relation to the implementation of clinical research results

Regarding research funders’ facilitative roles in relation to implementation two common roles for two different funding levels were identified: “Advocacy work” and “Monitoring implementation outcomes” (Table 3). “Advocacy work” was mentioned by one national public and three national private funders. All four defined this role in a similar manner, but worked with implementation-related issues from different standpoints: for instance, the national public funder stated that their role was to communicate among decision makers that implementation is important, whereas the national private funder attempted to convince decision makers to invest in implementation and also saw their role as educating the general public:

*“We have an evangelizing role that aims to educate the general public, the media and the politicians about the development [in our focus field] and the resources and expenses that are demanded to implement the results”.*

(-Funder from the national private level)

Two national private and one local public funder considered that “Monitoring implementation outcomes”, i.e. to evaluate whether the implemented clinical research results improved health outcomes and saved costs, is an important part of their work in relation to implementation. The national private funders had a structured way to monitor implementation outcomes either through a yearly published report describing the research results and their implementation or through an updated register about treatments that had been implemented and the outcomes for patients. The local public funder in turn stated that it is their primary assignment to monitor implementation outcomes, although no mention of how this is performed was made:

*“Yes, research that does not lead to any result in healthcare, improvement of care methods, new drugs, new treatments or new diagnose forms and so forth, what is the meaning with that? I think it is the primary task for us to monitor this [implementation outcomes]”.*

(-Funder from the local public level)

All in all, the 10 funders mentioned eight facilitative roles in relation to implementation. Regarding the roles within the funding levels, the national public funders selected three different roles which all were supported by one funder. The local public funders indicated two different roles supported by one funder each, whereas the national private funders mentioned five different roles, two of which were supported by all three funders – “Advocacy work” and “Dissemination of knowledge” – and one which was supported by two out of three funders, “Monitoring implementation outcomes” (Table 3). “Dissemination of knowledge,” a role indicated by all three private funders, was not recognized by any of the other two funding levels. All three private funders described “Dissemination of knowledge” in similar terms, as something that goes beyond sending out newsletters and publishing results on the internet:

*“We organize that kind of conferences [to inform and discuss research results and their possible implementation] and we invite speakers which are skillful in these issues [implementation and the clinical areas]”.*

(-Funder from the national private level)

## Who is responsible for implementation of clinical research results?

The funders identified six different actors responsible for implementation. Table 4 provides the list of these actors.

The “County Councils” (3 funders from 3 different levels), followed by the “Head of hospital units” (3 funders from 2 different levels) were the actors most often pinpointed as responsible for implementation. The key role of the County Councils was further underlined by the fact that one more funder viewed them as responsible *together with* other actors, namely medical practitioners. All in all, funders believed that the responsibility for implementation is located in the healthcare setting. As for the perceptions of the three groups of funders, national public funders agreed in general that it is the County Councils, alone or in concert with other actors, who are responsible for implementation. Similarly, national private funders leaned towards the County Councils and the “Head of hospital units” as responsible actors, with the exception of one private funder which stated that it is the research funders together with researchers who are responsible for implementation. Also, the local public funders considered that the County Councils or actors within the County Councils are responsible for implementation.

## Do the identified actors take responsibility for implementation of clinical research results?

As shown in Table 4, the majority of the three funders who considered that it is the County Councils who are responsible for implementation stated that County Councils take responsibility for implementation of clinical research results “To a certain degree” and only a minority felt that the County Councils do not take such responsibility. Among the funders who were slightly positive and considered that the County Councils take responsibility “To a certain degree” two alternative explanations were proposed: 1) the system through which clinical research results are transformed into practice is not really clear and there is room for improvement, and 2) the County Councils focus on saving lives and under economic pressure the County Councils do not focus on implementation of new findings:

*“Hospitals find themselves today under economic pressure and one understands that the usual care comes first as you must save lives – and that is not so peculiar – but there should be more space at certain hospitals which are university hospitals to do this [implement new findings]”.*

(-Funder from the local public level)

A more negative view had to do with the County Councils inability to take responsibility as expressed by the national private funder who considered that the County Councils are not organizationally capable of taking such responsibility:

*“County Councils [running the healthcare] are reactive not proactive, which means that they take the problems when they arise – so to say – they do not have a long-term planning”.*

(-Funder from the national private level)

The option “Head of hospital units” as responsible for implementation was supported by three funders from two different levels where the majority of them considered that the “Head of hospital units” take responsibility, whereas a minority stated that this actor takes responsibility “To a certain degree”. As for the different funding levels, all national public funders considered that the various actors they mentioned take responsibility for implementation “To a certain degree”, whereas the majority of the national private funders agreed that the mentioned funders take responsibility and only a minority stated that one of the mentioned actors do not take responsibility. On the other hand, half of the local public funders considered that the mentioned actors take responsibility, and half stated that the mentioned actors take responsibility “To a certain degree” (Table 4).

## Should someone else take responsibility for implementation of clinical research results?

Based on Table 5, half of the funders considered that *no-one else should take responsibility* for implementation, followed by three funders who thought that someone else should take responsibility “To a certain degree” and two funders who considered that *someone else* should take responsibility. National private funders were most convinced that no-one else should take responsibility, whereas both national and local public funders were more divided on this issue. On the national public level every funder had a differing opinion whereas the option “To a certain degree” was supported by half of the funders on the local public level and the “No” and “Yes” options received each support from one funder. National public funders mentioned actors varying from “Society” to “National coordination group”, whereas on the local public level the actors proposed were “Shared University/County Council organ”, “Biomedical center” and “Corporate group within the County Council”.
