# Supplementary material for: Research funders’ roles and perceived responsibilities in relation to the implementation of clinical research results: a multiple case study of Swedish research funders
Source: Implement Sci. 2015 Jul 17;10:100. doi: 10.1186/s13012-015-0290-5 (PMC4506440; doi:10.1186/s13012-015-0290-5)
Supplement: Additional file 5: — Detailed discussion of the findings. This file discusses more thoroughly the findings. [file 13012_2015_290_MOESM5_ESM.docx]

# Additional file 5. Detailed discussion of the findings

The discussion of the findings explicitly addresses the two purposes of this study: (1) identifying the facilitative roles of 10 Swedish clinical research funders in relation to the implementation of clinical research results; and (2) analyzing the funders’ views on implementation responsibilities.

## Research funders’ facilitative roles

All in all, the eight facilitative roles mentioned by the funders provide a nuanced picture of research funders’ facilitative roles in relation to implementation: from a *post-implementation* role expressed as “Monitoring implementation outcomes” to a *direct involvement* in implementation in terms of “Educate healthcare personnel and parents”, and “Work actively towards implementation”; and from *indirect support* via “Dissemination of knowledge”, “Create structures for organized introduction”, and “Advocacy work” to a *general and abstract support* such as “Create conditions for implementation through legislation in implementation related issues” and “Stimulate collaboration between researchers and industry”

Two of the identified roles were common for at least two different funding levels: “Advocacy work” and “Monitoring implementation outcomes”. “Advocacy work” is an acknowledged role for research funders aiming to facilitate implementation of research results through raising awareness and influencing for example universities to acknowledge merits that go beyond publication [1]. Creating awareness and increasing knowledge among decision makers at different levels regarding feasibility and costs of implementation was identified, in this study, as an advocatory activity. For “Monitoring implementation outcomes”, the second common role, the two national private funders even had an explicit structure for monitoring and effectiveness evaluation. This role identifies important activities for research funders occurring *after* implementation and has not been reported in previous research. In addition to these two common roles, a role shared by national private funders only, yet reported in previous research, was the “Dissemination of knowledge” [2, 3]. This role can be very simple, e.g. through sending out a newsletter, or more demanding, such as by organizing a seminar and inviting guest speakers to discuss the findings.

In general, many of the facilitative roles appear to fit the funders in question: the national public funders identify few facilitative roles (3), which fits their main mission of funding research; while national private funders assumed a greater number of facilitative roles (5) and especially roles related to indirect support such as “Advocacy work” and “Dissemination of knowledge”, which fits their mission of improving health. The local public funders are the units within the County Councils funding a significant amount of clinical research. However, due to the large size and complexity of their mother organization, the local public funders are not necessarily connected to the part of the County Council providing healthcare or organizing implementation. Consequently, the roles that local public funders perceive as theirs in relation to implementation may purposefully differ from the more explicit implementation roles of other healthcare-providing parts of the County Councils. This fact can explain the few facilitative roles (2) identified by the local public funders and their lack of involvement in activities such as “Advocacy work” and “Dissemination of knowledge”.

## Who is responsible?

The funders from all levels considered that the actors responsible for implementation are either the healthcare providing County Councils or some actors within the County Councils. In other words, there is a consensus among funders that the responsibility for implementation lies in the healthcare setting. This consensus reflects the Swedish law stating that the County Councils are required to deliver good quality care that is continually developed [4], whereby implementation of clinical research results can be viewed as part of this assignment. However, the fact that the three funding levels together proposed five different County Council-related actors as responsible for implementation may be a potential barrier for implementation. It suggests a “problem of many hands” whereby responsibilities become so unclear that implementation risks becoming no-one’s responsibility [5]. However, on the other hand the local public funders, which are closest to the healthcare setting, proposed three different actors as responsible for implementation not so much because of the “problem of many hands”, but because actors responsible for implementation can indeed be different from one County Council to another. In fact, County Councils are independent from the central government in how they assign implementation responsibility: for instance a County Council may have attributed implementation responsibility to hospital heads, whereas in another it might be less well-defined. In cases where the quite diffuse and large actors, such as “County Councils” or even more the “Healthcare system”, were identified as responsible the “problem of many hands” is a concrete risk due to the size and complexity of these organizations.

In terms of personal or organizational responsibility, half of the funders seemed to consider that implementation is a matter of *organizational* responsibility [6, 7]. In fact, “County Councils”, “Healthcare system,” and “The medical practitioners together with County Councils” were suggested by five funders. Instead, a pure personal responsibility [8, 9] within County Councils was attributed by three funders to the “Head of hospital units”. This form of personal responsibility is also a form of hierarchical responsibility, whereby the person highest in rank is made responsible [9, 10]. However, the heads of hospital units are merely managers and, following the argument of hierarchical responsibility [11], the real responsibility ultimately rests on the organizational leadership, usually taking the form of a group of actors. “Hospital leadership” as a category was supported by only one local public funder. Still, the prominence of organizational or shared responsibility is confirmed by the fact that four out of five categories regarding the actors related to County Councils refer to a group of actors or organizations, indicating a *collective and organizational* responsibility [12, 13].

## Do the actors take responsibility, or should someone else?

The funders in general were somewhat hesitant whether the actors identified take responsibility for implementation as five funders considered that the identified actors take responsibility “To a certain degree” and one funder reported that the identified actor does not take responsibility. The local public funders were most positive about responsibility taking, followed by the national public funders. The national private funders were divided over this (Table 4). As for the alternative responsible actors, considering all three levels of funders, half of the funders suggested that *no-one else* should take responsibility (Table 5). Also three funders supported the option of another responsible actor “To a certain degree”, which is somewhere between “No” and “Yes”. This result can be interpreted as the majority of funders being rather content with the identified actors. Regarding the alternative actors, the actors proposed by the local public funders were in general more concrete than the ones proposed by the national public funders. For instance “Shared University/County Council organ”, proposed by one local public funder, seems a more concrete option for taking responsibility for implementation than “Society”, as suggested by one national public funder.

# References

1. Holmes B, Scarrow G, Schellenberg M: **Translating evidence into practice: the role of health research funders.** *Implement Sci* 2012, **7**:39.

2. Lomas J: **Using “Linkage and Exchange” to Move Research into Policy at a Canadian Foundation**. *Health Aff* 2000, **19**:236–240.

3. Tetroe JM, Graham ID, Foy R, Robinson N, Eccles MP, Wensing M, Durieux P, Légaré F, Palmhøj Nielson C, Adily A, Ward JE, Porter C, Shea B, Grimshaw JM: **Health Research Funding Agencies’ Support and Promotion of Knowledge Translation: An International Study.** *Milbank Q* 2008, **86**:125–155.

4. Socialdepartementet: *Hälso- och sjukvårdslag SFS 1982:763*. Stockholm; 1982.

5. Thompson DF: **Moral Responsibility of Public Officials: The Problem of Many Hands**. *Am Polit Sci Rev* 1980, **74**:905–916.

6. Stone CD: *Where the Law Ends: The Social Control of Corporate Behavior.* New York: Harper & Row; 1975.

7. Braithwaite J, Makkai T: **Testing an Expected Utility Model of Corporate Deterrence**. *Law Soc Rev* 1991, **25**:7–40.

8. Peters BG: *The Politics of Bureaucracy.* 5th ed. London: Routledge; 2001.

9. Jackall R: *Moral Mazes: The World of Corporate Managers.* 20th ed. New York: Oxford University Press; 2010.

10. Coffee JC: **Beyond the Shut-Eyed Sentry: Toward a Theoretical View of Corporate Misconduct and an Effective Legal Response.** *Va Law Rev* 1977, **63**:1099–1278.

11. Bovens M: *The Quest for Responsibility. Accountability and Citizenship in Complex Organisations*. Cambridge: Cambridge University Press; 1998.

12. MacIntyre A: *After Virtue: A Study in Moral Theory*. 3rd ed. Notre Dame: University of Notre Dame Press; 2007.

13. Walsh WH: **Pride, Shame and Responsibility.** *Philos Q* 1970, **20**:1–13.

14. Miller GJ: **The Political Evolution of Principal-Agent Models.** *Annu Rev Polit Sci* 2005, **8**:203–225.

15. Waterman RW, Meier KJ: **Principal-Agent Models: An Expansion?**. *J Public Adm Res Theory* 1998, **8**:173–202.

16. Van Slyke DM: **Agents or Stewards: Using Theory to Understand the Government-Nonprofit Social Service Contracting Relationship**. *J Public Adm Res Theory* 2007, **17**:157–187.

17. Moe TM: **The New Economics of Organization**. *Am J Pol Sci* 1984, **28**:739–777.

18. Tomkins C: **Interdependencies, trust and information in relationships, alliances and networks**. *Accounting, Organ Soc* 2001, **26**:161–191.
